# Heterogeneous Nuclear Ribonucleoproteins: Implications in Neurological Diseases

**DOI:** 10.1007/s12035-020-02137-4

**Published:** 2020-09-30

**Authors:** Yi-Hua Low, Yasmine Asi, Sandrine C. Foti, Tammaryn Lashley

**Affiliations:** 1 The Queen Square Brain Bank for Neurological Disorders, Department of Clinical and Movement Disorders, UCL Queen Square Institute of Neurology, University College London, London, WC1N 3BG UK; 2 Duke-NUS Medical School, Singapore, Singapore; 3 Department of Neurodegenerative Disease, UCL Queen Square Institute of Neurology, University College London, London, UK

**Keywords:** hnRNPs, Alzheimer’s disease, Multiple sclerosis, ALS, FTD

## Abstract

Heterogenous nuclear ribonucleoproteins (hnRNPs) are a complex and functionally diverse family of RNA binding proteins with multifarious roles. They are involved, directly or indirectly, in alternative splicing, transcriptional and translational regulation, stress granule formation, cell cycle regulation, and axonal transport. It is unsurprising, given their heavy involvement in maintaining functional integrity of the cell, that their dysfunction has neurological implications. However, compared to their more established roles in cancer, the evidence of hnRNP implication in neurological diseases is still in its infancy. This review aims to consolidate the evidences for hnRNP involvement in neurological diseases, with a focus on spinal muscular atrophy (SMA), Alzheimer’s disease (AD), amyotrophic lateral sclerosis (ALS), frontotemporal dementia (FTD), multiple sclerosis (MS), congenital myasthenic syndrome (CMS), and fragile X-associated tremor/ataxia syndrome (FXTAS). Understanding more about hnRNP involvement in neurological diseases can further elucidate the pathomechanisms involved in these diseases and perhaps guide future therapeutic advances.

## Introduction

Heterogeneous nuclear ribonucleoproteins (hnRNPs) are a family of functionally diverse RNA bindings proteins (RBPs) [1]. Originally named alphabetically from A1 to U, they range from 34 to 120 kDA [2]. Their high involvement in RNA metabolic processes including pre-mRNA processing, splicing, and nucleocytoplasmic shuttling makes them pivotal in the regulation of gene expression [3]. Having a substantial control over post-transcriptional modifications and translation, it is unsurprising that aberrance in hnRNP function can lead to dire functional consequences. While their role in regulating several cellular processes is established, their role in neurological diseases has not been comprehensively investigated. This review aims to consolidate the existing literature of hnRNP abnormalities in various neurological diseases and spur further research in this area.

## Structure of hnRNPs

**Fig. 1:**
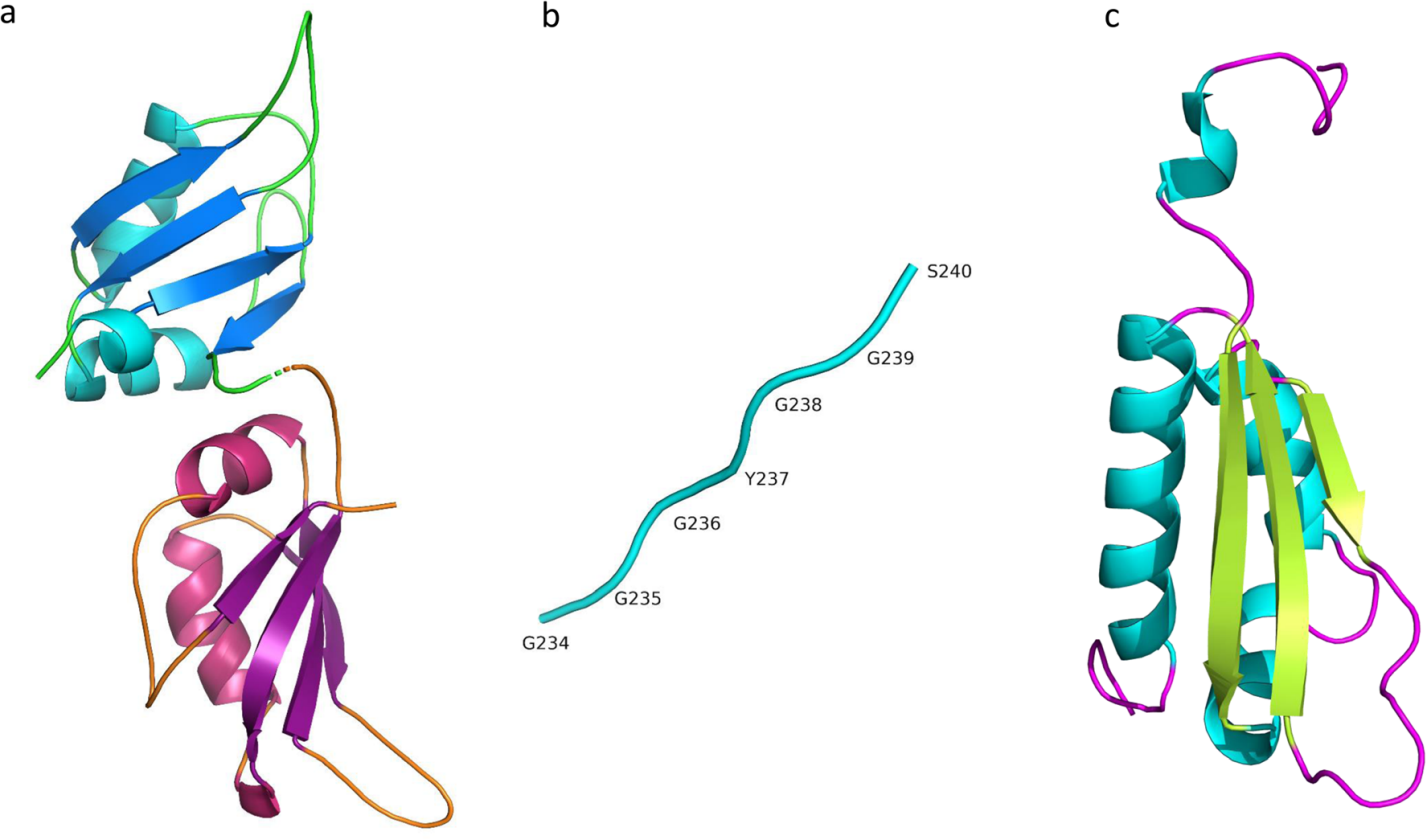
RNA binding domains in hnRNPs. RNA-binding domains in hnRNP include RRM (*RNA Recognition Motif)*, KH (*K-Homology*), and RGG (*Arginine-Glycine-Glycine)*. hnRNPA1 UP1, which spans the first 196 aa at the N-terminus, contains two RRM one in each subdomain of UP1. **a** RRM contains two α-helices (RRM1: cyan, RRM2: pink), four β-sheets (RRM1: blue, RRM2: purple), and five loops (RRM1: green, RRM2: orange) that order as βαββαβ. **b** The C-terminal of hnRNPA1 contains another RNA-binding motif known as RGG. The name reflects the abundance of Arg-Gly-Gly tripeptide repeats in the motif. **c** The KH domain of hnRNPK consists of three α-helices (cyan), three β-sheets (green), and five loops (purple) that fold in the order of βααββα [7–11]

Four evolutionary conserved RNA binding domains (RBD) have been elucidated in hnRNPs. The RNA recognition motif (RRM) is one of the most abundant protein domains in eukaryotes and was first discovered in the U1A protein [4]. It consists of 4 β-sheet (β4β1β3β2) and 2 α-helix (α1α2) domains which fold into a sandwich structure to bind RNAs [5]. Its two consensus sequences which are involved in RNA interaction, RNP1 (Lys/Arg-Gly-Phe/Tyr-Gly/Ala-Phe/Tyr-Val/Ile/Leu-X-Phe/Tyr) and RNP2 (Ile/Val/Leu-Phe/Tyr-Ile/Val/Leu-X-Asn-Leu), are located in β3 and β1 respectively [4]. These motifs, along with distinctive and varied terminal N- and C-sequences, account for the specific affinities of RNA binding [6] (Fig. 1).

Some hnRNPs contain human K (KH) domains in place of RBMs, named after hnRNP K in which they were first discovered [12]. Different KH domains bind a multitude of molecules; however, they all have a similar structure consisting of three anti-parallel β-strands and three α-helices (βααββα) with surface loops extending from the structure [13], enabling association to RNA molecules [12] (Fig. 1). Some hnRNPs also bind RNA via RGG domains, which are glycine-rich regions interspersed with arginine residues that are integral for RNA binding. The RGG domain also consists of aromatic rings which are responsible for hydrophobic interactions with RNA bases [14]. The glycine residues serve as hinges that allow the proteins to conform to a structure whereby arginine molecules or aromatic rings can come into contact with RNA [14] (Fig. 1).

HnRNPs also possess nuclear localisation sequences and/or nucleocytoplasmic shuttling domains. These ensure hnRNPs are both localized to the nucleus and allow them to be shuttled in order to undertake their cytoplasmic functions [3, 15]. While some hnRNPs can be shuttled in and out of the nucleus, others (hnRNP C and U) localize exclusively to the nucleus [16]. Since most hnRNPs are predominantly nuclear in their steady state and their function is highly dependent on their intracellular location, the integrity of the NLS is of paramount importance [17]. It is noteworthy that NLS defects have been linked to hnRNP instability and abnormal protein distribution [18, 19].

## Function of hnRNPs

### Gene Regulation

HnRNPs have the ability to both positively and negatively regulate gene expression depending on their binding partner. For instance, hnRNP A binds to the inhibitory subunit of nf-kb alpha, triggering the activation of transcription factor nf-kb [20]. HnRNP K interacts with the promotor of the *c-myc* gene via the pyrimidine-rich CT region in vivo and in vitro [21, 22] as well as the promotor of *eIF4E* which both promote transcription [23]. In addition, hnRNP K also binds to the TATA-binding protein and interacts with RNA polymerase II transcription machinery, thus stimulating transcription [24]. Conversely, hnRNP K can also suppress transcription by binding to the zinc finger transcriptional repressor which contains a KRAB-A domain involved in transcriptional repression [25]. Therefore, hnRNPs play key roles in gene regulation and it is likely that hnRNP misregulation may be involved in the pathomechanisms of multiple diseases.

### Alternative Splicing

Alternative splicing is a process which leads to great protein diversity and regulates gene expression [26] and the brain is particularly reliant compared to other regions [27, 28]. As a result, the pivotal role of hnRNPs in regulating gene expression and cell metabolism renders the integrity and health of the nervous system highly dependent on their function [29].

Abnormal splicing produces modified or defective proteins which may underlie neurological diseases due to the reliance of the CNS on specific isoforms for vital processes, such as long term potentiation and neurotransmission [30]. HnRNPs are heavily involved in alternative splicing as evidenced by its ability to globally regulate splicing in different cells and tissue types by regulating the availability of its IDR GY-rich motif [31]. Furthermore, iCLIP procedures have revealed 2394 clusters of hnRNP A2/B1 binding sites on 564 genes, including those involved in protein binding, myelination, and neurite projection [32], demonstrating its heavy role in alternative splicing. HnRNP A2/B1 binds to UAGG motifs in a regulatory manner [32], and the glycine-rich domain of hnRNP A1 is involved in splice-site silencing by looping of the RNA between binding elements [33, 34]. HnRNP C is able to crosslink to uridine tracts, regulating alternative splicing [35]. HnRNP F and hnRNP H bind RNA containing G quadruplexes and G tract motifs which result in alternate splicing [29, 36] and hnRNP A binds to splicing factor U2AF [37]. TDP-43, another member of the hnRNP family, is also found to be implicated in many neurodegenerative diseases. Its splicing roles include binding to UG rich regions, non-coding RNAs, 3’ UTRs of mRNAs, and deep intronic binding sites which results in silencing exon inclusion [33]. The critical roles of hnRNPs in mRNA processing and regulating cell metabolism strongly suggest that its aberrance may play a role in neurological diseases and this will be discussed in later sections.

### Stress Granule Formation

Stress granules form during periods of cellular stress as a form of energy preservation. The pooling of various RBPs and silenced mRNAs alters the overall levels of functional proteins and are thought to be crucibles of disease [38, 39]. In genetic abnormalities whereby there are expansion repeats, aberrant mRNA can also form toxic aggregates and consequently sequester important factors that regulate gene expression or apoptosis [40, 41]. Accordingly, several RBPs and signaling molecules such as hnRNP A, hnRNP B, TRAF2, RACK1, mTORC1, TDP-43, and FUS have been discovered to be sequestered in stress granules, thereby impairing their usual cell regulatory functions [42–47]. Apart from the loss of function due to its sequestration in stress granules, it has been proposed that hnRNPs are also involved in stress granule formation, and that stress granule assembly may be deficient with hnRNP abnormalities. Intrinsically disordered regions (IDR) within hnRNPs were found to be involved in liquid-liquid phase separation, a process which contributes to stress granule assembly [48]. Mutations in IDRs in hnRNP A1 have also been linked to ALS [49], suggesting deficient stress granule formation in ALS. This is an expanding concept and has previously been reviewed in depth [50–52].

### Cell Cycle Regulation

Often juxtaposed alongside cancer in epidemiological studies, cumulating evidence is beginning to elucidate that beneath the disparate cellular features of cancer and neurodegeneration lies a common underlying pathology of misregulated cell cycle events [53, 54]. For instance, cell death in Alzheimer’s disease (AD) has been postulated to be a result of abnormal cell cycle re-entry which mediates neuronal death [55]. Damaged neurons in the AD brain were also found to possess biomarkers of cell cycle events [56]. Furthermore, persisting genomic instability, possibly due to replication stress and the misregulation of nucleic acid binding proteins, can trigger cellular death [57]. However, despite the roles of hnRNP in cell cycle regulation, much is still unknown about their roles in cell death. For instance, HnRNP A1 is known to be involved in telomere maintenance [58, 59], and its misregulation may result in premature cell death. HnRNP K, a putative cell cycle regulator that interacts with p53 and c-myc and causes cell death and excitotoxicity respectively when misregulated [53, 60], has been disproportionately studied more in cancer and its role in neurodegeneration is still unclear.

### Axonal Transport

Due to the unique extensive morphology of neurons and their high dependence on intact intracellular protein transport, axonal defects are often thought to precede neurodegeneration [61–64]. Neurons also possess intricate and expansive transcriptomic profiles with its axons containing hundreds to thousands of mRNAs, including mRNAs of proteins that regulate gene expression in response to local axonal activity [65]. In addition to the main transport machinery such as dynein, kinesin, and their cargos, RBPs also play an indispensable supportive role in maintaining axonal transport. For instance, hnRNP R co-localizes with beta-actin mRNA in axons and its knockdown impaired axonal growth [66]. Furthermore, hnRNP R interacts with 7SK, a non-coding RNA involved in axon elongation, and reduced levels of hnRNP R depleted 7SK levels and consequently impaired axon growth [67]. HnRNP K was also found to interact with the transcripts of several cytoskeletal genes such as *Arp2*, *tau*, and *α-internexin-like-neurofilament*, all of which are integral for axonogenesis and intracellular transport [68]. HnRNPs are also involved in regulating specific axonal translation. For instance, hnRNP A2/B1 was found to interact with Netrin-1 DCC, which binds a subset of mRNAs involved in cell-cell adhesion and protein targeting, inducing the translation of specific subsets of mRNAs [69, 70]. HnRNP H1, H2, and F are also involved in regulating axonal mRNA regulation [71]. It was found that a combined knockdown reduced levels of axonal *Hmgb1* and *Nrn1* mRNA levels, and decreased axonal protein synthesis [71]. With the diverse roles of hnRNPs in maintaining neuronal health and the dire consequences when perturbed, it is reasonable to postulate that neurodegeneration can be precipitated by the misregulation of hnRNP in the neuronal parenchyma.

## HnRNPs in Neurological Diseases

Mounting evidence points to RNA perturbations as the underlying driving factor of neurodegenerative diseases such as ALS and FTD [72–74]. In light of this, there has been a major paradigm shift away from targeting abnormal protein accumulation in neurodegeneration, towards investigating possible RNA disturbances as a precipitating factor for neurological pathogenesis [75] (Table 1). There is emerging evidence of an overlapping RNA pathology in otherwise distinct neurodegenerative diseases, and therefore there is a need to further investigate hnRNPs which tightly govern RNA processing. This may be a hopeful avenue for the development of future effective therapeutic targets.

**Table 1:** Neurological diseases where hnRNPs have been implicated in the disease process. Highlighting additional hnRNP proteins identified not including the primary protiens found in the pathological inclusions, for example TDP-43 and FUS in FTLD and ALS

Disease	hnRNP	Function	Reference
Spinal muscular atrophy (SMA)	hnRNP G	Splicing	Hofmann & Wirth 2002 [79]
	hnRNP Q	Splicing	Chen et al. 2008 [81]
	hnRNP M	Splicing	Cho et al. 2014 [82]
	hnRNP A1	Splicing	Kashima et al. 2007 [84] Koed Doktor et al. 2011 [85] Baek et al. 2019 [86]
	hnRNP R	Transport of protein	Rossoll et al. 2003 [93]
Alzheimer’s disease (AD)	hnRNP A1	Splicing	Donev et al. 2007 [97] Bekenstein & Soreq 2013 [98]
	hnRNP C	Translation	Lee et al. 2010 [108]
	hnRNP Q	Protein folding and aggregation	Ashraf, Ganash & Athanasios, 2019 [113]
Amyotrophic lateral sclerosis (ALS) and frontotemporal dementia (FTD)	hnRNP H and F	Binding to expansion repeats	Lee et al. 2013 [138]
	hnRNP A1	Present in pathological inclusions	Honda et al. 2015 [150] Gami-Patel et al. 2016 [152]
	hnRNP A3	Present in pathological inclusions	Mori K et al. 2013 [137]
	hnRNP E2	Present in pathological inclusions	Davidson et al. 2017 [151]
	hnRNP D	Present in pathological inclusions	Gami-Patel et al. 2016 [152]
	hnRNP G	Present in pathological inclusions	Gami-Patel et al. 2016 [152]
	hnRNP I	Present in pathological inclusions	Gami-Patel et al. 2016 [152]
	hnRNP L	Present in pathological inclusions	Gami-Patel et al. 2016 [152]
	hnRNP Q	Present in pathological inclusions	Gittings et al. 2019 [153]
	hnRNP R	Present in pathological inclusions	Gittings et al. 2019 [153]
Multiple sclerosis (MS)	hnRNP A1	Nucleocytoplasmic transport	Lee & Levin 2014 [163]
	hnRNP H	Exon skipping	Paraboschi et al. 2014 [168]
Congenital myasthenic syndrome (CMS)	hnRNP H	Splicing	Ohno et al. 2017 [170]
	hnRNP L	Splicing	Rahman et al. 2013 [173]
Fragile X-associated tremor/ataxia syndrome (FXTAS)	hnRNP A2	Binding to repeats	Sofola et al. 2007 [184]

### Spinal Muscular Atrophy

**Fig. 2:**
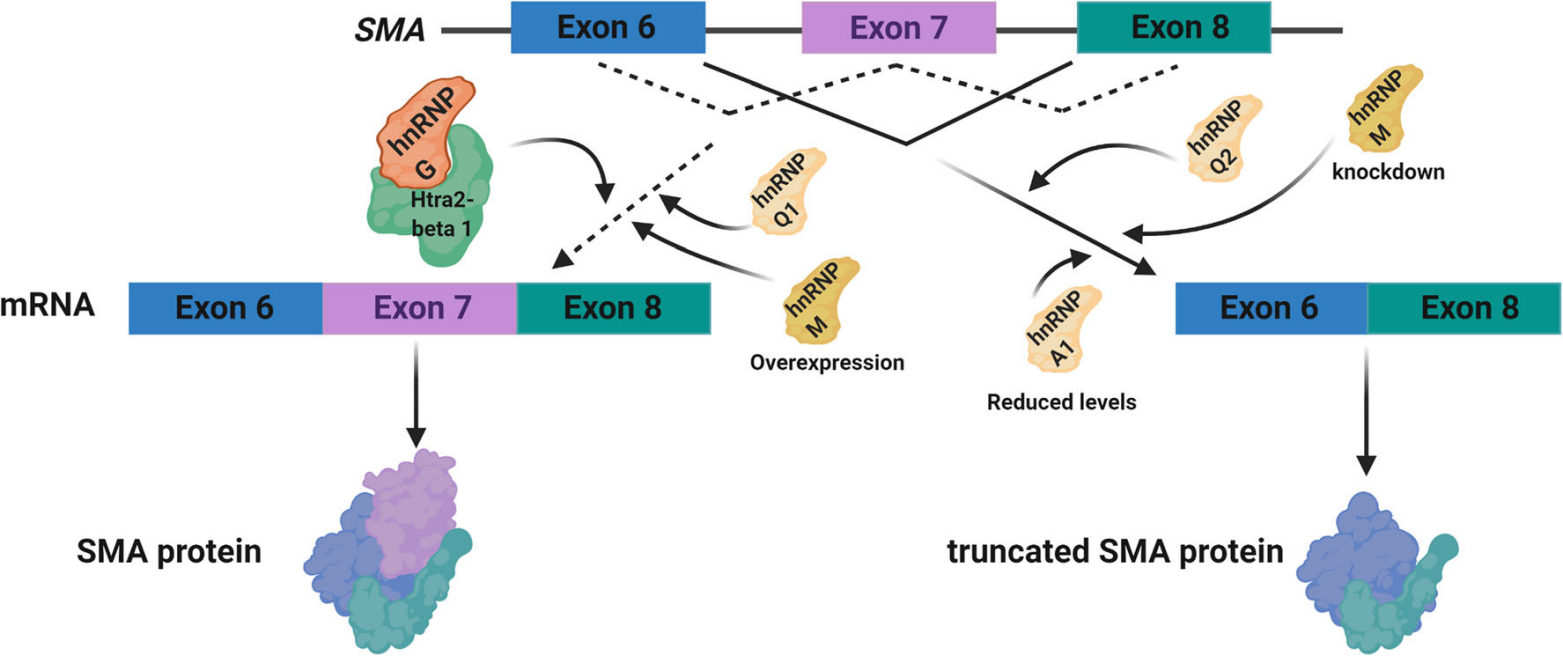
hnRNPs involvement in spinal muscular atrophy (SMA). Various hnRNPs have been implicated in regulating splicing of the *SMA* gene. Promoting the inclusion of exon 7 to produce a functional SMA protein or the exclusion of exon 7 which forms a truncated SMA protein.

Spinal muscular atrophy (SMA) is an autosomal recessive disease marked by motor neuron death in the anterior horn of the spinal cord. The SMN protein, a protein integral for motor neuron survival, is encoded by the *SMN1* gene which is found to be deleted or mutated in SMA [76]. Despite a deficient *SMN1*, SMA patients possess a paralogous duplicate gene known as *SMN2*, a gene almost identical to *SMN1* except that it excludes exon 7 in its splicing. This transcript produces a truncated non-functional SMN protein [77], resulting in a failure to confer sufficient rescue. As a result, identifying RBPs that regulate the splicing of *SMN2* in order to promote the inclusion of exon 7 has been extensively studied as a potential therapeutic target for SMA [78] (Fig. 2). It has been shown that several RBPs regulate the splicing of exon 7, suggesting a possible misregulation of these proteins as a contributor to SMA. For instance, hnRNP G was found to promote the inclusion of exon 7 via direct interaction with Tra2-β1, a splicing factor which induces exon 7 inclusion [79, 80]. In mice, it was also found that hnRNP Q could modulate the splicing inclusion and exclusion of exon 7 depending on the ratio of its isoforms, with overexpression of major isoform Q1 promoting inclusion by direct binding to exon 7 [81]. In addition, not only was hnRNP M overexpression found to facilitate exon 7 inclusion in patient cells through binding an enhancer on exon 7 via recruitment of U2AF65, its knockdown also fostered a splicing environment which excluded exon 7 [82]. However, HnRNP A1 seems to have opposing effects. For instance, C6T transitions in *SMN2* genes have been found to create a high affinity exonic silencer binding site for splicing repressor hnRNP A1 [83]. While some postulate high specificity in hnRNP A1 splicing repressor [84], others propose a more general inhibitory role of hnRNP A1 by binding to a common ESS motif spanning the 3′ splice site of exons [85]. It was found that reduction in hnRNP A1 levels correlated with inclusion of exon 7, and a resultant increase in SMN protein [86]. Despite the uncertainty in the precise mechanism of hnRNP A1 repression which warrants further investigation, it is apparent that hnRNP A1 promotes the exclusion of exon 7. One proposed mechanism is that both RRMs in hnRNP A1 bind to the human intronic splicing silencer ISS-N1 as structural disruptions to either or both RRMs can successfully impair exon 7 splicing repression [87].

Apart from splicing regulation, it has been suggested that hnRNPs could also have roles in transporting the SMN protein which is fundamental for neuronal health. While it has been established that SMN proteins are crucial in axonal growth and local synaptic action [88, 89], however, less attention has been placed on the accompanying molecular partners behind it. HnRNP R was found to co-localize with SMN in axons as well as in presynaptic terminals both in vivo and in vitro [90, 91]. HnRNP R also facilitates complex formation with β-actin mRNA [66, 92], suggesting that apart from regulating splicing, hnRNPs also facilitates transport of SMN proteins. Corroborating this, it was found that overexpression of hnRNP R encourages neurite outgrowth in PC12 cells, and that hnRNP R is needed for the binding of SMN to β-actin mRNA [93]. These findings indicate that perturbations to normal hnRNP levels may compromise axonal transport and could serve as another route for neurodegeneration considering the heavy dependence of neurons on intact intracellular transport for localized protein synthesis [66, 92].

Recent advances in the therapeutics of SMA have led to the development of a novel drug Nusinersen, also known as Spinraza, which promotes the expression of full length SMN2 [94–96]. Nusinersen is a modified antisense oligonucleotide which binds to the SMN2 pre-messenger RNA, encouraging the inclusion of exon 7, and augmenting the production of functional SMN protein which is deficient in SMA [94–96]. Phase 3 trials have found that patients who were on Nusinersen had significant improvements of motor functions as compared to the control group [95]. Clinical success through manipulating the splicing pathway in SMA makes hnRNPs a promising target of therapeutic research with its heavy role in alternative splicing.

### Alzheimer’s Disease

AD is a debilitating neurodegenerative disease characterized by neuronal death and abnormal protein aggregates comprising of Aβ and hyperphosphorylated neurofibrillary tau tangles. The initial links between hnRNPs and AD surfaced when it was discovered that hnRNP A1 modulates the splicing of the *APP* gene, which is the precursor of the Aβ peptide [97, 98]. It was demonstrated that an imbalance in APP isoforms, in particular high concentrations of APP770, potentially elevates Aβ secretion. HnRNP A1, working in concert with SC35, alternatively splices the APP pre-mRNA by binding to hexanucelotide repeat expansions (HREs) within Alu and flanking the introns adjacent to exons 7, thus generating APP695 which is protective against Aβ toxicity [97]. This suggests that increasing hnRNP A levels may ameliorate AD pathology whereas decreased levels may be associated with worsened pathology and the progression of sporadic AD [99].

However, while some evidence suggests a neuroprotective role of elevated levels of hnRNP A1, others present contradictory results. For instance, apart from managing alternative splicing of *APP*, hnRNP A1 also regulates balance of receptor for advanced glycation end products (RAGE) isoforms mRAGE and esRAGE [100]. It was found that increased relative concentrations of mRAGE, which is linked to AD pathology, were induced by an overexpression of hnRNP A1 [100]. Higher levels of hnRNP A1 are also expressed in peripheral blood mononuclear cells in patients with AD, suggesting that the misregulation of hnRNP A1 along with decreased levels of transcription factor miR-590-3p may be associated with neuronal death [101].

Another angle of neurodegeneration adopts a prion like perspective in which abnormal protein aggregates act like prionoids and exhibit properties such as templating and incessant proliferation [102–105]. AD is known to be a notorious proteinopathy, and HnRNP A1 was proposed to exhibit prion like properties [106] and may be in part responsible for intercellular prion like spread of proteins resulting diseases such as AD, ALS, and FTD [107].

Unlike the unequivocal role of hnRNP A1 in modulating the pathology of AD, the roles of other hnRNPs are less clear. hnRNP C has been found to promote *APP* translation by competing with the *FMRP* gene for the same region [108] and also by stabilizing the APP precursor mRNA by interacting with the 29 nt element at the 3′-untranslated regions [109], suggesting that increased hnRNP C levels promote Aβ secretion. On the other hand, it was found that cAMP regulated APP processing in mechanisms independent of hnRNP C [110]. In addition, a decrease of hnRNP B1 has been shown in the hippocampal regions of AD patients [111], whereas hnRNP B1 levels are relatively preserved in the inferior temporal cortex [112]. Though the mechanism is unclear, hnRNP Q lnRNAs have been found to be crucial in regulating protein folding and aggregation, and were found to be associated with AD [113].

Despite the expanding idea that AD is associated with abnormal re-entry to the cell cycle and that hnRNPs are pivotal in regulating cell cycle events [55, 114–116], there has been an underappreciation of the potential underlying link between hnRNP abnormalities and its modulation of cell cycle events and neuronal death. P53, a major cell cycle regulator, gates cells at the G0/G1 and G2/Gm phases and is crucial in maintaining balance between proliferation and apoptosis [53] and hnRNP K is also a necessary cofactor of this regulation [117]. Furthermore, in times of DNA stress and damage, which is typical in AD [118], hnRNP K sumoylation mediates the regulatory mechanisms of p53 [115]. Not only is hnRNP K one of the most important regulators of p53 action, it was discovered earlier that p53 levels are elevated in the temporal cortices of AD patients [119]; hence, it is surprising that hnRNP K misregulation has not been investigated in AD and further suggests a possible hnRNP related pathway of neurodegeneration.

### Amyotrophic Lateral Sclerosis and Frontotemporal Dementia

Amyotrophic lateral sclerosis (ALS) is the most common form of motor neuron disease, marked by significant muscle wasting and widespread neurodegeneration of both upper and lower motor neurons. Clinically, patients exhibit spinal symptoms of muscular weakness, spasticity, fasciculations, fatigue and bulbar symptoms of dysphagia, and breathing difficulties [120]. In contrast, frontotemporal dementia (FTD) is characterized by progressive neurodegeneration in the frontal and temporal cortices with accompanying changes in personality and language abilities [121]. Phenotypic heterogeneity is observed in ALS and FTD patients, specifically where ubiquinated TDP-43 pathologies are found [72]. Although pathogenic variants in *TARDBP* develop TDP-43 pathologies, other familial and sporadic ALS cases also develop TDP-43 pathologies [122]. However, despite the rarity of *TARDBP* mutations in FTD, those with TDP-43 pathologies account for around 45% of FTD cases [123]. TDP-43 is a DNA/RNA binding protein which is usually concentrated in the nucleus [124]. It contains a nuclear localization signal and nuclear export signal and hence is able to shuttle between the nucleus and cytoplasm [124]. The known functions of TDP-43 are broad, including regulating gene expression and involvement in several RNA processing steps such as pre-mRNA splicing, regulation of mRNA stability, mRNA transport, translation, and the regulation of non-coding RNAs [33, 125, 126]. Notably, the majority of *TARDBP* mutations are located in the glycine-rich region at the carboxy-terminal region, which is also the region that interacts with other hnRNPs and is heavily involved in pre-mRNA splicing regulation [127]. Pathologically, TDP-43 accumulates in the cytoplasm resulting in a loss of nuclear TDP-43, and it is this loss from the nucleus that has led to proposed mechanisms of disease involving a loss of normal function in the nucleus, a toxic gain of function in the cytoplasm, or both. These mechanisms have been tested in many animal models [128–133]. As TDP-43 homeostasis is crucial for normal cellular function, increasing evidence suggests that aberrant TDP-43 regulation may result in disease. Excess TDP-43 in the cytoplasm may lead to formation of inclusion bodies resulting in cellular dysfunction, while nuclear depletion may induce widespread dysregulation of mRNA metabolism, with TDP-43 knockdown shown to cause differential splicing or expression of hundreds of targets [134–136].

**Fig. 3:**
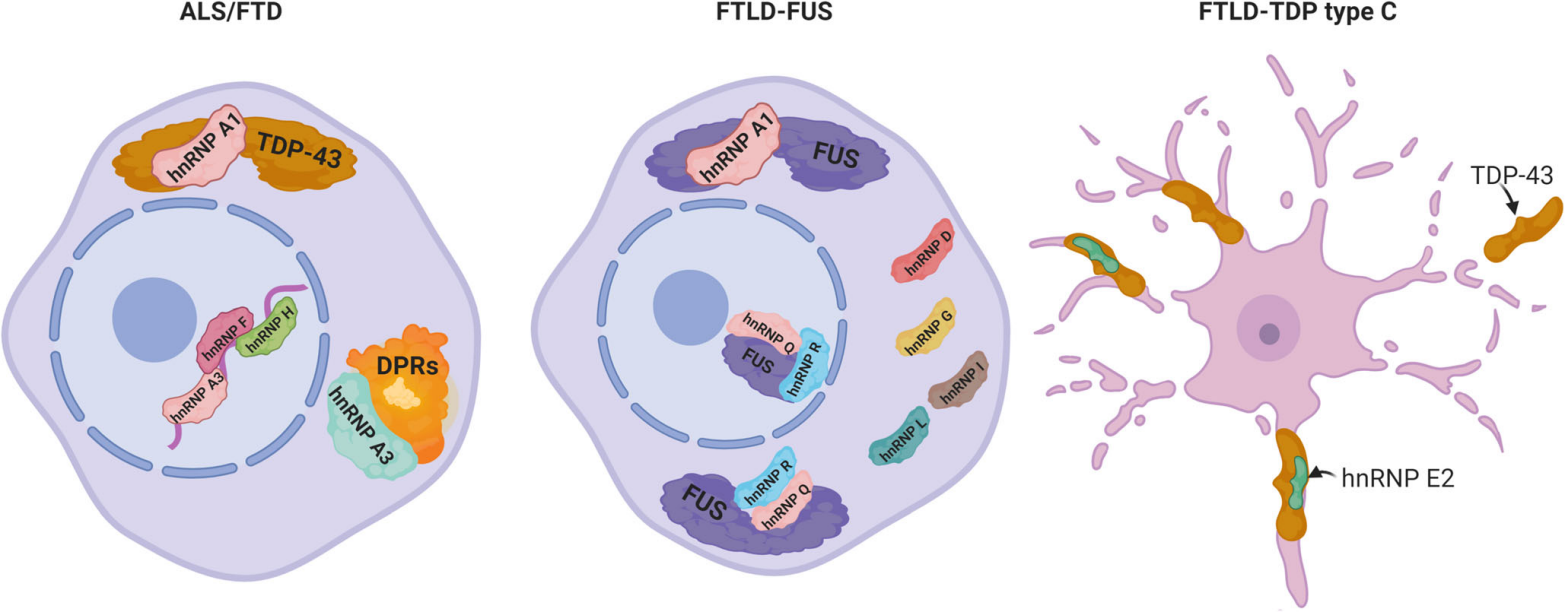
The involvement of hnRNPs in amyotrophic lateral sclerosis and frontotemporal dementia. hnRNPs have been shown to be involved in ALS and FTD in many ways. hnRNPs are able to bind to the hexanucleotide repeats within the nucleus, bind to the dipeptide repeat protein (DPRs) inclusions in the cytoplasm and also to the main pathological inclusions in both diseases TDP-43. In FTLD-FUS, hnRNP proteins have been found to co-localize with FUS in the nucleus and the cytoplasm. It has also been shown that other hnRNP proteins also form patholigcal inclusions without the presence of FUS. FTLD-TDP type C pathology shown distinct inclusions as long twisted neurites which have also been shown to co-localize with hnRNP E2

The most common genetic cause of ALS and FTD is *C9orf72* expansions which produce extensive amounts of GGGGCC repeats [74]. It has been shown that GGGGCC expansions can become highly neurotoxic, correlating with disease severity and are able to sequester otherwise functional RBPs. HnRNP A3 has been identified in neuronal cytoplasmic and intranuclear inclusions in patients with GGGGCC expansion repeats [137] (Fig. 3). HnRNP H and hnRNP F were also found to co-localize with GGGGCC expansion foci in immunoprecipitation studies [138] and western blot analyses [139] respectively. It has been recently suggested that a GGA-rich sequence on hnRNP H may account for its affinity to the GGGGCC expansion repeats and its sequestration in ALS and FTD [140]. HnRNP sequestration by G quadruplexes in the expansion repeats in ALS and FTD suggests that hnRNP implication may be in part responsible for the toxicity incurred by *C9orf72* mutations as important RNA processes such as splicing are compromised [141]. In addition, TDP-43, has also been found to be regulated by, co-localize with, or interact with hnRNPs A1, A2/B1, C1/C2, A3, K [127, 142, 143], suggesting a strong association of impaired hnRNP translation machinery and neurological diseases (Fig. 3). Apart from the ability of GGGGCC repeats to sequester important proteins, several other pathogenic mechanisms have been suggested, including loss of function of C9orf72 and generation of toxic dipeptide repeats [144, 145], and these have been reviewed in detail elsewhere [146, 147].

As hnRNPs are normally localized to the nucleus, cytoplasmic redistribution has been linked to disease pathology and impaired nucleocytoplasmic transport has been suggested to be a common feature of ALS and FTD [49, 148]. For instance, mutations in hnRNP A1 can disrupt its nuclear localization sequence and result in cytoplasmic redistribution and aggregation [149]. It was also found in ALS patients that hnRNP A1, along with TDP-43, had a greater tendency to aggregate in the cytoplasmic inclusions compared to controls [150]. Another hnRNP, E2, also co-localized with TDP-43 in pathological inclusions of the semantic dementia subtype FTD patients [151] (Fig. 3). In FTLD FUS-positive patients, hnRNP A1, D, G, I, and L, which are involved in nuclear transport, have been found to exhibit pathological mislocalisation or accumulated in neuronal cytoplasmic inclusions [152] (Fig. 3). hnRNPs R and Q have also been found to co-deposit with FUS in FTLD-FUS inclusions [153], suggesting that impaired nucleocytoplasmic transport may contribute to disease pathology (Fig. 3).

As in AD, it has also been suggested that ALS proteins such as TDP-43, SOD1, and FUS, exhibit a prion like spread [154]. Accordingly, it was revealed that mutations in the prion like domains in the *hnRNP A1* and *A2/B1* genes increased the propensity for protein self-aggregation, excessive, fibrilisation, and incorporation into stress granules [155]. Furthermore, it is thought that even though cytoplasmic formation of membrane-less organelles such as stress granules may be advantageous for energy conservation, high concentrations of hnRNPs aggregated within stress granules may accelerate fibrillisation [49].

### Multiple Sclerosis

Multiple sclerosis (MS) is an autoimmune disorder of the central nervous system characterized by demyelination; however, the exact target epitope of autoimmunity remains unclear [156]. Focus has been placed on autoimmunity against myelin related targets and it has been shown that antibodies against myelin proteins such as MOG and MBP are not specific to MS and are also elevated in other neurological diseases [157]. As a result, there has been a growing interest in investigating non-myelin targets in contributing to the pathogenesis of MS [158]. HnRNP A1 and hnRNP B1 are highly expressed in neurons [159, 160], and were found at significantly high levels in the CSF of MS patients than in other diseases [161]. Interestingly, their antibodies were found in MS patients. The antibodies generated may have dual roles as they can on one hand impair proper functioning of epitopes and on the other hand directly damage the cells. For instance, MS patients developed antibodies against the M9 epitope of hnRNP A1 where the nuclear localization sequence is found [162, 163]. In line with this, MS patients typically possess genomic single nucleotide variants in the *hnRNP A1* gene within the nucleocytoplasmic transport domain TPNO-1, suggesting that impaired hnRNP A1 mediated nucleocytoplasmic transport could be involved in MS pathology [163]. On the other hand, the antibodies generated can also cause a toxic gain of function. It was found that anti-hnRNP A1 antibodies penetrate neuronal cells and alter levels of ATP and apoptotic regulator caspase 3/7, as well as promote a redistribution of hnRNP A1 intracellularly [164]. They also alter transcripts related to hnRNP A1 function, reduce neuronal processes and distort the cytoplasm [162]. Compared to hnRNP A1, less is known about hnRNP B1 despite the elevations of its antibodies in MS patients. Not only were hnRNP B1 antibodies found in the CSF of a large proportion of MS patients examined, the generation of hnRNP B1 antibodies in the CSF was found to be specific only to MS and not found in other neurological diseases [165]. Although not much is known about the precise implications brought about by hnRNP B1 antibodies, its specificity to MS and its potential as a biomarker provides ample basis for further research.

There is also evidence suggesting that hnRNP A1 may be responsible for the phenotypical features in MS. It was found that hnRNP A1 binds to *spastin*, a gene responsible for hereditary spastic paraparesis, which produces a phenotype that is closely similar to MS [162]. This finding was also replicated by another group which found a hnRNP A1 antibodies in animal models also contributed to neurodegeneration as evidenced by increased localization to stress granules, worsened experimental autoimmune encephalomyelitis (EAE) cases, modifications in phenotype from flaccid to spastic paralysis, and selective degeneration in the cerebellar white matter [166].

Aside from being possible epitopic targets, hnRNPs may also mediate ectopic splicing, causing alterations to levels of RNA and proteins. Lower levels of *PRKCA* have been shown to increase MS susceptibility [167]. Interestingly, it was found that hnRNP H overexpression mediates the expression of the *PRKCA* gene by promoting the skipping of exon 3 while hnRNP H silenced cells increased exon 3 inclusion [168], suggesting that hnRNP H abnormalities may be related to MS predisposition.

### Congenital Myasthenic Syndrome

Congenital myasthenic syndrome (CMS) consists of genetically inherited diseases in which normal neurotransmission is impaired at the neuromuscular endplate. CMS usually stems from mutations in the muscle nicotinic acetyl choline receptors (AChR) subunits, AChE deficiencies, or inefficient kinetics of AChRs [169]. As in other neurological diseases, the roles of hnRNPs in regulating alternative splicing are pivotal in maintaining transcripts necessary for healthy functioning. A variety of molecules are expressed at the neuromuscular junction and its abnormal expression, highly regulated by RBPs, is impaired in CMS.

The alpha subunit of AChR is encoded by the *CHRNA1*, and it was discovered that hnRNP H binds to an intronic splicing silencer on *CHRNA1* and enhances the skipping of a non-functional P3A exon. The exclusive inclusion of exon P3A is often displayed in CMS causing genetic mutations [170]. From individual patients with CMS, it was revealed that G > A mutations in this region led to a 100-fold decrease in the binding affinity of hnRNP H, suggesting a regulatory role of the alternative splicing of exon P3A [171]. It was later also discovered that a tannic acid induced increase in polypyrimidine tract binding protein could rescue the effects of hnRNP H mediated inclusion by binding close intron 3 on *CHRNA1*, promoting its exclusion [172]. HnRNP L and hnRNP LL also antagonistically bind the polypyrimidine tract binding protein which modulates P3A splicing. It was found that mutated *CHRNA1* generates an otherwise absent binding site for hnRNP LL, a splicing enhancer, and results in the displacement of hnRNP L, a splicing suppressor, which subsequently leads to exclusive P3A inclusion, a pathological feature that is found in CMS [173].

Not only does hnRNP H regulate *CHRNA1*, it also was found to be involved in the regulation of AChE_T_, AChE_H_, and AChE_R_, with the former two being expressed at neuromuscular junctions and hematopoietic cells respectively, and the third being rarely expressed [170]. HnRNP H competes against another splice site regulator CstF64 and suppresses cryptic PAS, thereby generating AChE_T_ [170]. CstF64 has opposing effects which activates cryptic PAS and its overpowering of hnRNP H generates the other two subtypes AChE_H_ and AChE_R_ [170]. This suggests that abnormal hnRNP H activity may result in alterations in AChE levels at the neuromuscular endplate, another feature of CMS.

Another molecule critical in CMS is the muscle specific receptor tyrosine kinase (MuSK), which is involved in the pre-patterning of AChRs, a process required for the high AChR concentration in neuromuscular junctions [174, 175]. The *MuSK* gene consists of a frizzled cysteine-rich region that is essential for both proper folding as well as the binding of Wnt ligands, important for mediating AChR clustering [176–178]. Interestingly, exon 10 of the *MuSK* gene, which encodes 6 out of 10 of the essential cysteines, is alternatively spliced and functionally skipped in humans but not in mouse. It was revealed that hnRNP C regulates the skipping of exon 10 by binding to the poly-T tract in the exon splicing silencer 5 region, and also mediates hnRNP L and YB-1 binding leading to further additive effects [179].

Collagen Q, encoded by the *COLQ* gene, is necessary for the anchoring of AChE to NMJ endplates [180] and mutations in *COLQ* are associated with AChE deficiency, a defect often evident in CMS [181]. HnRNP H suppresses the splicing of exon 16 in *COLQ* and results in aberrant skipping, and is antagonistically modulated by SRSF1, a splicing enhancer that promotes its inclusion [180, 182].

Whereas the exact roles of P3A and exon 10 alternative splicing have yet to be elucidated, the disease-causing effects of the missplicing of these proteins which are subjected to hnRNP regulation provide a strong basis for further investigation.

### Fragile X-Associated Tremor/Ataxia Syndrome

Fragile X-associated tremor/ataxia syndrome *(*FXTAS) is an adult onset neurodegenerative disorder and has phenotypic characteristics that include motor and cognitive impairment. It is predominant in males and involves the expansion of CGG repeats in the fragile X mental retardation gene *FMR1* [183]. As in other neurological disorders with expansion repeats, the CGG repeats sequester important RBPs, thus depriving the cell of the normal function of these RBPs and thereby contributing to the pathomechanisms of the disease. HnRNP A2 binds to the CGG repeats in fly models [184] and is found in cytoplasmic inclusions in FXTAS post mortem brains [185]. Furthermore, it was found that hnRNP A2 overexpression could suppress the rough eye phenotype caused by rCGG [184], suggesting that rCGG sequestration of hnRNP A2 may be involved in the pathogenesis of FXTAS since its over expression could rescue the neurodegenerative phenotype. It was also found that misspliced hnRNP A2/B1, which is prevalent in CGG expression, could be corrected by TDP-43 and resulted in a corresponding reduction of rCGG induced damage [186].

Not only does sequestration of hnRNP A2 diminish its normal cellular functions, it was also revealed that hnRNP A2 can mediate neurodegeneration through interaction with its binding partners. It was shown that miRNA miR-277 could mediate CGG neurodegeneration as overexpression of miR-277 increased neuronal toxicity whereas decreased expression of miR-277 suppressed neurodegeneration [187]. Coincidently, miR-277 is also regulated by hnRNP A2 [187], suggesting that sequestration of hnRNP A2 by CGG repeats could further result in ectopic miR-277 levels, resulting in a loop which further perpetuates rCGG mediated neurodegeneration. The neurodegenerative effects of rCGG are mediated by retrotransposon activation, and it was discovered that hnRNP A2 has the ability to suppress the toxicity of a particular retrotransposon, Gypsy, by binding to HP1, a retrotransposon silencer [188].

It has also been suggested that the CGG repeats are constantly expanding dynamic structures and may recruit more proteins at later stages. For instance, it was found that in later stages of the disease where CGG aggregates have expanded significantly, sam-68 mediated hnRNP G inclusions were also found [189]. This suggest that the rCGG sequences themselves may not recruit proteins directly, but does so indirectly by protein-protein interactions with inclusions that were recruited earlier. This is noteworthy since hnRNPs are known to have an expanding repertoire of binding partners; hence, more information about hnRNP mediated sequestration of other proteins into inclusion bodies may shed light onto the pathomechanisms of disease.

## Conclusion

This review provides a summary of the existing evidence of aberrant hnRNP profiles in various neurological diseases, specifically SMA, AD, ALS, FTD, MS, CMS, and FXTAS. Our understanding of hnRNPs in these diseases is still in its infancy, and there remains much that has yet to be uncovered. Human genome studies have revealed that 40–60% of our genome relies extensively on alternative splicing to confer functional diversification upon our otherwise limited gene repertoire [190]. With the viability and normal functioning of cells being heavily subjected to intact and tightly regulated splicing mechanisms, it would be expected that RBPs dysfunction can serve as major contributors to disease pathology [191].

However, the array of hnRNP mediated interactions, as well as binding partners, are far from being fully elucidated, and better understanding in the structural morphology of hnRNPs and their interacting partners may provide further insight into their roles in health and disease. Its implication in various neurological diseases highly suggests that hnRNPs possess an underappreciated role in disease pathology. The role of hnRNPs in neurological disease has been a largely overlooked area and more research may serve as a promising platform for the development of novel therapeutic targets.
